# The association between CD2+ peripheral blood lymphocyte subsets and the relapse of bladder cancer in prophylactically BCG-treated patients

**DOI:** 10.1038/sj.bjc.6690185

**Published:** 1999-03

**Authors:** E Reyes, J Carballido, L Manzano, L Moltó, C Olivier, M Alvarez-Mon

**Affiliations:** Department of Medicine, Service of Immune System Diseases/Oncology, University Hospital ‘Principe de Asturias’, University of Alcalá, Madrid, Spain; Department of Urology, Clinic ‘Puerta de Hierro’, Madrid, Spain

**Keywords:** bladder neoplasm, BCG, immunotherapy, T-lymphocytes, recurrence

## Abstract

We investigated the potential existence of differences in the distribution of T-lymphocyte subsets and in the proliferative response of these CD2+ cells to polyclonal mitogens in patients with transitional cell bladder carcinoma (SBTCC) treated with prophylactic intracavitary instillations of bacillus Calmette–Guérin (BCG) according to their clinical response to this treatment. Before BCG treatment, different subset distribution (CD8+ and CD3+ CD56+), activation antigen expression (CD3+ HLA– DR+) and proliferative response to mitogenic signals were found in CD2+ cells from SBTCC patients prophylactically treated with BCG who remained free of disease or those who had recurrence of tumour. Otherwise, the prophylactic intracavitary BCG instillations in SBTCC patients are associated with a transitory variation of T-lymphocyte subset distribution (CD4 and CD8) and activation antigens expression (CD25). © 1999 Cancer Research Campaign


*British Journal of Cancer* (1999) **79**(7/8), 1162–1167
© 1999 Cancer Research Campaign
Article no. bjoc.1998.0185

### The association between CD2+ peripheral blood lymphocyte subsets and the relapse of bladder cancer in prophylactically BCG-treated patients

**E Reyes[1], J Carballido[2], L Manzano[1], L Moltó[1], C Olivier[2] and M Alvarez-Mon[1]**

[1]Department of Medicine, Service of Immune System Diseases/Oncology, University Hospital 'Principe de Asturias', University of Alcalá, Madrid, Spain;
[2]Department of Urology, Clinic 'Puerta de Hierro', Madrid, Spain

**Summary** We investigated the potential existence of differences in the distribution of T-lymphocyte subsets and in the proliferative response of these CD2+ cells to polyclonal mitogens in patients with transitional cell bladder carcinoma (SBTCC) treated with prophylactic intracavitary instillations of bacillus Calmette–Guérin (BCG) according to their clinical response to this treatment. Before BCG treatment, different subset distribution (CD8+ and CD3+ CD56+), activation antigen expression (CD3+ HLA– DR+) and proliferative response to mitogenic signals were found in CD2+ cells from SBTCC patients prophylactically treated with BCG who remained free of disease or those who had recurrence of tumour. Otherwise, the prophylactic intracavitary BCG instillations in SBTCC patients are associated with a transitory variation of T-lymphocyte subset distribution (CD4 and CD8) and activation antigens expression (CD25).

**Keywords:** bladder neoplasm; BCG; immunotherapy; T-lymphocytes; recurrence

Superficial tumours are the most frequent presentation of transitional cell carcinoma of the bladder (SBTCC) (Pansadoro et al, 1995; Rosenbaum et al, 1996). These tumours demonstrate a higher tendency to recur either at the same stage and grade or as deeply invasive tumours after initial surgical resection (Hurle et al, 1996; Lamm and Torti, 1996; Esrig et al, 1997). This biological behaviour constitutes a common clinical dilemma as far as an optimal management and points to the need for an effective prophylactic treatment of these tumours (Carballido et al, 1990*a*; Kurth, 1997; Kurth et al, 1997; Moltó et al, 1997; Witjes, 1997) after their transurethral resection of the tumor (Patard et al, 1993; Molto et al, 1995).

Interestingly, several alterations have been described in the immune system of patients with SBTCC. These alterations include the functional impairment of T-lymphocytes and NK cells, whose intensity appears to be related to the progression of the disease (Carballido et al, 1990*a*; 1990*b*; Jackson et al, 1994).

Intravesical instillation of several immunomodulators, such as bacillus Calmette–Guérin (BCG), has been proposed as prophylactic adjuvant therapy (Erton et al, 1996; Kurth, 1997b; Witjes, 1997). The mechanism of action of intracavitary instillations with BCG remains partially known. A potential direct effect of BCG against tumour cells inhibiting their proliferation and inducing their differentiation has been proposed (Patard et al, 1993; Jackson et al, 1994; Akaza et al, 1995). There is also evidence that intracavitary BCG instillations induce a strong infiltration of inflammatory cells including activated immune cells in the bladder wall (Maes et al, 1995; Erton et al, 1996). The characteristics of this immune response appears to be related to the clinical outcome of the patients (Ratliff et al, 1993; Zlotta et al, 1994; Maes et al, 1995; De Reijke et al, 1996; Turner and Dockrell, 1996; Moltó et al, 1997). The immunoregulatory effects of intracavitary BCG instillations in SBTCC patients may play a relevant role in its anti-tumour effect.

Heterogeneous clinical response to intracavitary BCG instillations in SBTCC patients have been described (Nadler et al, 1994; Mack and Frick, 1995; O'Donnell and DeWolf, 1995; Takashi et al, 1995). There is evidence that around 30% of treated patients show recurrence of the disease (Nadler et al, 1994; Lapham et al, 1997). It has been shown that the patient's immune system status may determine the response to the treatment with immunomodulators (Hermann et al, 1990; Verhagen et al, 1990; Moltó et al, 1997). It is possible that the phenotypical and functional characteristics of the immune system of untreated SBTCC patients after transurethral resection of the tumour may have a prognostic tool for the identification of the clinical response to prophylactic use of BCG.

We have investigated the potential existence of differences in the distribution of T-lymphocyte subsets and in the proliferative response of these CD2+ cells to polyclonal mitogens in patients with SBTCC treated with prophylactic intracavitary instillations with BCG, in accordance with their clinical response to this treatment. In those patients who remained free of recurrence of the disease after 1 year of follow-up, we also studied the effects of intracavitary instillations with BCG in these immunological parameters.

*Received 7 October 1997*
*Revised 20 April 1998*
*Accepted 30 June 1998*

*Correspondence to:* M Alvarez-Mon, Department of Medicine, University of Alcala de Henares, Carretera Madrid-Barcelona, Km 33,600 28871 Alcala de Henares, Madrid, Spain

### MATERIALS AND METHODS

### Patients

Eighteen patients with histologically proven transitional cell bladder carcinoma were included in this study (Table 1). The degree of tumour invasion was classified according to the tumor

**Table 1** Patient and tumour characteristics[a]

| | |
|---|---|
| Age (years) | 64 (41–82) |
| Sex | 18 Male: 0 Female |
| Number of tumours | |
| Single | 12 |
| Multiple | 6 |
| Size | |
| ≤2 cm | 4 |
| >2 cm | 14 |
| Stage | |
| Ta (multiple) | 2 |
| T1 | 16 |
| Grade | |
| 1 | 0 |
| 2 | 7 |
| 3 | 11 |

[a]All tumours were primary. Associated carcinoma in situ excluded.

nodes and metastasis staging system (TNM) adapted by the International Union Against Cancer, and grade according to Koss (1973). All the patients entered in the study had superficial tumours (stages Ta and T1 and grades 1 to 3). The tumours were completely resected with the muscle layer in each case and random multiple biopsies were taken. None of the patients had received any treatment within the 6 months prior to the study, nor had they any other disease which could affect the immune system. Twenty healthy male control subjects (median 60 years and range 43–76) were selected for the study. All patients were studied prior to transurethral resection of the tumour and 3, 6 and 12 months after beginning a treatment according to the following protocol: 6 weekly intracavitary instillations of 81 mg of BCG (ImmuCyst, Connaught, Toronto, Canada). Five patients had recurrence during this follow-up period (median of recurrence time: 7 months, range 3–8) and had included in others protocols. Blood samples were obtained before the surgery and anaesthetic procedures and informed patient consent was secured for the study.

### Cell separation

Peripheral blood mononuclear cells were obtained by Ficoll–Hypaque (Lymphoprep Nyegaard and Co., Oslo, Norway) gradient (Boyum, 1968). T-cells were purified by rosetting with sheep red blood cells pretreated with 2-aminoiethylisothiouronium bromide, as previously described (Madsen et al, 1980). After counting, cells were resuspended in RPMI-1640 (Whitaker Bioproducts, Walkersville, USA) supplemented with 10% heat-inactivated fetal bovine serum (Biochrom KG, Berlin), L-glutamine (2 mM Biochrom KG, Berlin), Hepes (25 mM Biochrom), and 1% penicillin–streptomycin (Difco Lab, Detroit, MI, USA); this will be referred to as complete medium. Cell viability checked by trypan blue exclusion was always greater than 95%. The purity of phenotypically defined CD2+ lymphocytes in the rosette cellular preparations was higher than 85% in all cases. Cellular cultures were performed at 37°C in a humidified atmosphere containing 5% carbon dioxide.

### Staining of cells and flow cytometry analysis

Immunofluorescence studies were performed on fresh purified CD2+ lymphocyte preparations. In the dual-colour experiments, fluorescein isothiocyanate (FITC)- and expression of phycoerythrin (PE)-labelled MAbs against membrane antigens was analysed. The MAbs used in this study were anti-CD2 (T and NK cells), anti-CD56 (NK cells), anti-CD19 (B cells), anti-CD16 (NK cells), anti-CD4 (helper/inducer cells), anti-CD8 (cytotoxic/suppressor cells), anti-CD25 (α-chain of IL-2 receptor), anti-HLA-DR DR (MHC class II molecules), anti-CD71 (transferrin receptor), anti-CD45 (pan human leucocyte) and anti-CD14 (macrophages, neutrophils). Furthermore, the MAb anti-CD3 (T cells) was used as a third colour (PerCP). Control studies with unstained cells and cells incubated with isotype-matched irrelevant FITC-, PE- and PerCP-labelled MAbs were performed for each experiment. All MAbs were obtained from Becton and Dickinson (Mountain View, CA, USA). Acquisition and tricolour immunofluorescence analysis was performed with a FACScan flow cytometer and Lysis II software (Becton Dickinson). For leucocyte immunological study, a biparametric electronic gate was drawn around CD45 + CD14– cells.

### Proliferation studies

Fresh CD2+ lymphocytes (50 000 cells per well) were cultured in complete medium on 96 flat-bottom culture plates in the presence of different mitogens for 3, 5 and 7 days. The mitogenic stimuli analysed included phytohaemagglutinin (PHA, 10 μg $ml^{-1}$, Difco) in the presence or absence of recombinant human interleukin 2 (rhIL-2, 100 IU $ml^{-1}$, Hoffman-La Roche, Nutley, NJ, USA), plastic-immobilized anti-OKT3 (anti-CD3, 5 μg $ml^{-1}$, Ortho-mune, Orthodiagnostic System) and *O*-tetredecanoylphorbol 13-acetate (TPA, 10 ng $ml^{-1}$, Sigma, St Louis, MO, USA). DNA synthesis was measured during the last 18 h of the culture period by incubation of 1 μCi of [$^3$H]thymidine (Radiochemical Center, Amersham, UK).

### Statistical analysis

Comparison of the measures for the immune phenotype and proliferation median values between patients with or without recurrence and healthy controls were tested with the Mann–Whitney *U*-test. Differences in the same parameters in the SBTCC patient group who were free of tumour recurrence at the different times of follow-up were assessed with two related samples: a non-parametric test for repeated measurements. *P*-values were considered significant when less than 0.05. Analysis of data was done using SPSS for Windows (Release 6.0).

### RESULTS

### Different subset distribution and activation antigen expression were found in CD2+ cells from SBTCC patients prophylactically treated with BCG who remained free of disease or those who had recurrence of tumour

The potential existence of differences in the lymphocyte subset distribution and activation antigen expression of purified CD2+ cells from a group of 18 SBTCC patients before the prophylactic intracavitary instillations with BCG and healthy control subjects (*n* = 20) were analysed (Table 2). The SBTCC patients were classified according to their clinical performance after the intracavitary BCG treatment (13 patients remained without clinical and pathological evidence of tumour recurrence and five patients with recurrence introduced into other adjuvant protocols). The expression of the cell-surface antigens CD3, CD4, CD8, CD16, CD56

© Cancer Research Campaign 1999

**Table 2** Lymphocyte subpopulations and activation antigen expression before BCG treatment in patients who had no recurrence at the end of the study; those with relapse and healthy controls

| | SBTCC patients | | |
|---|---|---|---|
| MAb | Tumour recurrence (*n* = 5) | Free of tumour recurrence (*n* = 13) | Healthy controls (*n* = 20) |
| CD2+ | 88.2 ±3.0 | 90.9 ± 5.9 | 92.8 ± 3.2 |
| CD3+ | 72.2 ± 1.8 | 78.8 ± 3.5 | 76.9 ± 1.6 |
| CD4+ | 32.6 ± 7.5 | 39.5 ± 3.4 | 44.1 ± 2.0 |
| CD8+ | 41.0 ± 4.5[a,b] | 37.3 ± 2.9 | 37.2 ± 2.1 |
| CD4/CD8 | 0.9 ± 0.2[a] | 1.2 ± 0.1 | 1.3 ± 0.6 |
| CD16+ | 21.8 ± 6.4 | 19.4 ± 10.6 | 16.6 ± 8.9 |
| CD56+ | 29.4 ± 8.7 | 24.4 ± 11.6 | 23.2 ± 10.7 |
| CD3+CD56+ | 16.5 ± 7.7[a,b] | 8.3 ± 1.8 | 8.9 ± 1.4 |
| CD19+ | 2.7 ± 1.2 | 3.7 ± 3.4 | 2.8 ± 0.7 |
| CD25+ | 11.3 ± 0.9[a] | 17.3 ± 4.9[a] | 8.0 ± 1.1 |
| CD71+ | 2.7 ± 1.8[a,b] | 7.6 ± 3.2[a] | 3.8 ± 0.8 |
| CD3+HLADR+ | 5.6 ± 1.0[b] | 12.4 ± 4.8[a] | 5.7 ± 0.7 |

The values are expressed as means of percentage ± s.d. [a]$P<0.05$ vs. controls. [b]$P<0.05$ vs. patients with no recurrence.

and CD19 as well as activation antigens CD25, CD71 and HLA-DR was analysed in the purified CD2+ lymphocyte preparations from both groups of patients and healthy control subjects. There were no significant differences in the percentages of CD2+ lymphocyte purification obtained in the cellular preparations from the different groups of patients. There were no significant differences in the level of CD3+ lymphocytes and CD16+ NK cells present in the purified CD2+ fractions from both groups of SBTCC patients and healthy control subjects. However, the percentage of CD3+CD56+ cytotoxic T-lymphocytes was significantly higher in the CD2+ preparations from patients with recurrence of disease than in those found in patients who remained free of disease and healthy control subjects (Table 2).

There were no significant differences in the percentages of CD4 T-lymphocytes found in the CD2+ cells from both groups of patients and healthy controls (Table 2). However, the percentages of CD8+ lymphocytes in CD2+ preparations from patients with tumour recurrences were marginally but significantly higher than those found in patients who remained free of disease and healthy controls. The CD4/CD8 ratio was significantly lower in patients with tumour recurrences than that found in patients who remained free of disease and healthy controls.

The expression of CD25 antigen by CD2+ cells from healthy donors was lower than that in both groups of patients; however, no differences were observed between patient groups. In contrast, the expression of other activation antigens, namely CD71 and HLA-DR, was significantly higher in patients without recurrence compared with patients who relapsed or with healthy controls.

### Different proliferative response to mitogenic signals is found in CD2+ cells from SBTCC patients prophylactically treated with BCG who remained free of disease or those who had tumour recurrence

The proliferative response of CD2+ cells from the described SBTCC patients and healthy controls to polyclonal mitogens (PHA, anti-CD3 and TPA) in the presence or absence of rhIL-2 were analysed. In kinetic studies performed at 3, 5 and 7 days of culture, the maximal proliferative response to PHA of purified CD2+ preparations from patients and controls was found at 5 days of culture (data not shown). The proliferative response of CD2+ cells from patients with tumour recurrence to PHA stimulation in the presence or absence of exogenous rhIL-2 was significantly lower than that found in patients who remained free of disease (Figure 1). The proliferative response of CD2+ cells from both groups of SBTCC patients to rhIL-2 or PHA or PHA plus IL-2 was significantly decreased with respect to that found in healthy control subjects. There were no significant differences in the proliferative response of CD2+ cells in both groups of patients to the stimulation with anti-CD3 or TPA (Figure 1).

### The prophylactic intracavitary BCG instillations in SBTCC patients are associated with a transitory T-lymphocyte subset distribution and activation antigens expression

The potential existence of modifications in the distribution of the lymphocyte subsets in the CD2+ cell preparations from the SBTCC patients that completed the 12 months of follow-up (13 patients then remained free of tumour recurrence) after beginning of the intracavitary BCG instillations was analysed (Table 3).

At four different timepoints analysed during the 12 months of follow-up, there were no significant differences in the percentages of cells that expressed the CD2, CD3, CD16, CD56 or CD19 antigens in the purified CD2+ preparations from this group of SBTCC patients. However, the percentage of CD4 T-lymphocytes in CD2+ preparations increased significantly at 3 and 6 months after the beginning of the intracavitary BCG instillations with respect to the values observed before this treatment. A concomitant decrease in the percentage of CD8+ lymphocytes and increase in the CD4/CD8 ratio in those cellular preparations was observed. At 12 months of follow-up, these significant differences in the distribution of the CD4+ and CD8+ lymphocyte subsets in CD2+ preparations from the intracavitary BCG-treated patients disappeared, contrasting with those observed before this treatment.

The percentage expression of the activation antigen CD25 by CD2+ cells from these SBTCC patients also significantly increased after 6 months follow-up compared with that observed

© Cancer Research Campaign 1999

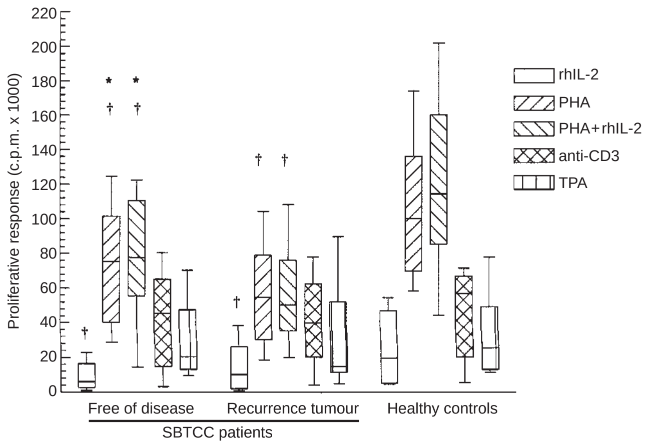

**Figure 1** Proliferative response to PHA, IL-2, PHA plus IL-2, anti-CD3 and TPA of CD2+ cells purified from peripheral blood before BCG treatment of the SBTCC patients (5 and 13 in the recurrence/no recurrence groups) respectively and healthy controls (*n* = 20). Cells, 50 000 per well were cultured in complete medium in the presence of different mitogens. Cultures were incubated at 37°C in a humidified atmosphere containing 5% carbon dioxide for 5 days. DNA synthesis was measured during the last 18 h of the culture period by incubation of 1 μCi [$^3$H]thymidine. Boxes represent the interquartile range (percentile 25 to percentile 75) of each group and the lines dividing the boxes represent the median in counts per minute (c.p.m.) of each group. Error bars represent percentile 5 and 95 respectively. **P*<0.05 vs. SBTCC patients with recurrence tumour. †*P*<0.05 vs. healthy controls after 3 months of the beginning of the intracavitary treatment (Table 3). There were no significant differences in the expression of CD71 antigen and HLA-DR molecules by these CD2+ cells at different times analysed throughout the study.

The proliferative response of CD2+ cells from these prophylactic intracavitary BCG-treated SBTCC patients to PHA, anti-CD3 antibodies and TPA during the 12 months of follow-up was analysed. In these patients, as well as in the healthy controls studied in parallel, we did not observe significant modifications in the expression of the antigens analysed in CD2+ cells or in their proliferative response to the polyclonal mitogens (data not shown).

### DISCUSSION

The mechanism of action of the intracavitary prophylactic instillations with BCG after transurethral resection of the tumour has yet to be established (Patard et al, 1993; O'Donnell and DeWolf, 1995). It is known that the effect of the biological response modifiers in the treatment of tumour patients are related to the patient's immune status. Although the patient groups included in this pilot study are small, our data suggest that those who remained free of tumour recurrence at 1 year follow-up after the initiation of the intracavitary instillations with BCG showed different phenotypic and functional characteristics in their purified CD2+ cells compared with those showing clinical and pathological evidence of tumour recurrence during the time of analysis. It is important to note that the tumour recurrence patients were included in different therapy protocols. For this reason, similar analysis could not be found in the non-responsive patient population.

An enhancement in the percentage of CD8 and CD56 T-lymphocytes in CD2+ cells before the BCG treatment was observed in patients with recurrence of the tumour when compared with those patients who remained free of disease. It is known that these T-lymphocyte subsets have been associated to suppressor/cytotoxic activities (Walker et al, 1995; Turner and Dockrell, 1996). Interestingly, it has been described that in the presence of IL-2 and IL-12, the CD3+CD56+ lymphocytes increase their cytotoxic activity and for this reason they may play an important positive role in the anti-tumour response of the patient's immune system (Satoh et al, 1996). This apparent contradiction might be related to a potential primary or secondary functional impairment of these effector cells and/or to a lack of sensitivity of the recurrent tumour cells to their lytic activity. It is also important to emphasize that the percentage of CD8 lymphocytes found in CD2+ cells from patients before BCG treatment who suffered recurrence of the disease during the follow-up was marginally but significantly higher than that found in healthy controls. This phenotypical abnormality found in CD2+ cells from patients with recurrence of the tumour is also associated with a significantly decreased proliferative response to polyclonal mitogenic stimulation with PHA. This blastogenic response to PHA is dependent on in vivo T-cell activation (HLA-DR expression)

**Table 3** Lymphocyte populations and expression of activation antigens in CD2+ cells purified from peripheral blood of SBTCC patients free of tumour recurrence at different times of follow-up

| | SBTCC patients (*n* = 13) | | | |
|---|---|---|---|---|
| **MAb** | **Basal** | **3 months** | **6 months** | **12 months** |
| CD2+ | 90.9 ± 5.9 | 90.7 ± 5.1 | 96.5 ± 4.0 | 93.1 ± 5.0 |
| CD3+ | 78.8 ± 3.5 | 74.1 ± 11.3 | 81.6 ± 4.9 | 76.3 ± 5.3 |
| CD4+ | 39.5 ± 3.4 | 51.2 ± 5.0[a] | 56.1 ± 2.3[a] | 49.7 ± 6.7 |
| CD8+ | 37.3 ± 2.9 | 24.6 ± 3.2[a] | 26.8 ± 5.3[a] | 33.3 ± 5.3 |
| CD4/CD8 | 1.2 ± 0.1 | 2.0 ± 0.9[a] | 2.1 ± 0.7[a] | 1.5 ± 0.6 |
| CD16+ | 19.4 ± 10.6 | 10.4 ± 2.3 | 11.8 ± 3.0 | 11.9 ± 2.3 |
| CD56+ | 24.4 ± 11.6 | 18.8 ± 6.1 | 20.7 ± 5.0 | 24.3 ± 5.3 |
| CD3+CD56+ | 8.3 ± 1.8 | 3.8 ± 0.9 | 6.3 ± 0.7 | 6.4 ± 1.0 |
| CD19+ | 3.7 ± 3.4 | 5.3 ± 2.6 | 5.3 ± 3.7 | 2.4 ± 2.5 |
| CD25+ | 17.3 ± 4.9 | 14.3 ± 3.6 | 22.6 ± 9.1[b] | 13.7 ± 6.3 |
| CD71+ | 7.6 ± 3.2 | 6.6 ± 2.1 | 7.0 ± 1.6 | 2.6 ± 0.4 |
| CD3+HLADR+ | 12.4 ± 4.8 | 11.2 ± 2.4 | 13.1 ± 4.7 | 9.8 ± 2.7 |

The values are expressed as means of percentage ± S.D. [a]*P*<0.05 vs. basal determination. [b]*P*<0.05 vs. determination at 3 and 12 months.

*© Cancer Research Campaign 1999*

(Turner and Dockrell, 1996) and on the use of the IL-2 regulatory pathways by them (Larson et al, 1980). It has been shown that CD8 lymphocytes may suppress the proliferative response of the whole population of T lymphocytes to IL-2 (Nicks et al, 1990). It is important to note that the defective proliferative response of CD2+ cells from patients with later recurrence of the disease to PHA is not reversed by exogenous IL-2. This severe defective proliferative response of CD2+ cells to PHA stimuli found in subjects with recurrence of the tumour, agrees with the observed clinical prognostic value of the induction of IL-2 messenger RNA on peripheral blood mononuclear cells from SBTCC patients treated with prophylactic intravesical BCG instillations (Kaempfer et al, 1996). The potential relevance of the IL-2 pathway in the response to BCG in these patients might also be suggested by the increased expression of the α-chain of the IL-2 receptor observed in CD2+ cells from patients that remain free of tumour recurrence after this prophylactic treatment.

Interestingly, before starting the intracavitary BCG instillations, CD2+ lymphocytes from patients who remained free of tumour recurrence during 12 months of follow-up showed a significant increased percentage of cells that expressed CD71 activation antigen and DR molecules with respect to those observed in patients with recurrence of the disease. It is known that the presence of tumour cells induce a response in the host's immune system. In this sense, CD2+ cells from both groups of patients have a clear percentage increase of activated T lymphocytes with respect to that found in healthy controls. Taken together, these results suggest that the existence of an in vivo activation of the CD2+ cells may act as a preconditional factor responsible for the clinical effectiveness of BCG. According to the data from this study, phenotypic and functional characteristics of CD2+ cells from patients with SBTCC are associated with different clinical responses to the prophylactic intracavitary BCG treatment. These results agree with previous findings in animal models that confirm the pivotal role of T lymphocytes and NK cells in BCG-mediated anti-tumour activity (Carballido et al, 1990*b*; Pryor et al, 1995; Thanhauser et al, 1995; Turner and Dockrell, 1996).

There is evidence that intravesical BCG induces a strong immune response in the bladder wall (Maes et al, 1995; De Reijke et al, 1996). Infiltration of activated CD4+ and CD8+ T lymphocytes and macrophages in the bladder wall appears to be related to the therapeutic activity of BCG (Pryor et al, 1995). The immune system is a complex cellular and molecular net with multiple and continuous functional interaction among its elements. Furthermore, there is also a continuous traffic of immune cells between organs and peripheral blood (Picker et al, 1990). In agreement with this physiological concept of the immune system, it has been shown that organ-localized inflammatory autoimmune diseases are associated with systemic immune system abnormalities (Maes et al, 1995). Accordingly, we have investigated the potential systemic effects of intracavitary instillations with BCG on the CD2+ cells of SBTCC patients. We have observed that the intracavitary instillations with BCG induce a significant enhancement in the percentage of the CD4+ T lymphocytes with a concomitant significant decrease in the CD8+ subsets. In addition, an increase in the expression of the CD25 lymphocytes by these CD2+ cells is also observed. These effects are observed at 3 and 6 months after the initiation of the six weekly instillations of BCG. The existence of these systemic modulatory effects of the intracavitary instillations with BCG agree with previous observations of functional effects of cytokine production by peripheral blood monocytes associated with this treatment (Jackson et al, 1995; Pryor et al, 1995). These systemic effects uphold the claim of functional lymphocyte interactions between peripheral blood lymphocytes and those involved in the local inflammatory bladder wall response (Prescott et al, 1992; Erton et al, 1996). A traffic of these bladder wall lymphocytes to peripheral blood may also be involved.

We have not observed that the defective proliferative response of CD2+ cells to polyclonal mitogens found before BCG treatment in those patients who remained free of disease would be normalized by this immunomodulatory treatment. This finding might suggest that modifications in the schedule of BCG treatment and/or association with other immunomodulatory agents may more effectively regulate the host's immune system and improve clinical results. Furthermore, the limited duration of the immunomodulator effect of intracavitary instillations with BCG indicates that the cyclic use of these bacteriological preparations might maintain the effect and improve therapeutic results of prophylactic use of intracavitary BCG in patients with SBTCC. The small number of patients included in this study limits the power of statistical test. Significance tests are being used to indicate particular areas that are identified for further investigation. Additional studies are required in order to define the clinical significance of the initial immune status of patients with SBTCC found in this pilot study. A prophylactic use of intracavitary instillations with BCG in patients with higher chance of positive response to this treatment might improve the clinical outcome.

### ACKNOWLEDGEMENTS

We would like to thank Ms María del Puerto Hernández for her linguistic assistance and T González, J Alvarez and J Sánchez from the 'Instituto Nacional de Investigaciones Agrarias' (El Encín, Alcalá de Henares, Madrid) for generously providing sheep red blood cells. This work was partially supported by grant 94-0023-01 from the 'Fondo de Investigación y Comisión Interministerial de Ciencia y Tecnología' of Spain.

### REFERENCES

Akaza H, Hinotsu S, Aso Y, Kakizoe T and Koiso K (1995) Bacillus Calmette-Guerin treatment of existing papillary bladder cancer and carcinoma in situ of the bladder. Four-year results. The Bladder Cancer BCG Study Group. *Cancer* **75**: 552–559

Boyum AJ (1968) Isolation of mononuclear cells and granulocytes from human blood. *Scan J Clin Lab Invest* **21**: 77–79

Carballido J, Alvarez Mon M, Olivier C and Molto L (1990*a*) Immunotherapy of urothelial neoplasms. Experimental and clinical implications of alpha interferon. *Arch Esp Urol* **43**: 149–158

Carballido J, Alvarez Mon M, Solovera OJ, Menendez Ondina L and Durantez A (1990*b*) Clinical significance of natural killer activity in patients with transitional cell carcinoma of the bladder. *J Urol* **143**: 29–33

De Reijke TM, De Boer EC, Kurth KH and Schamhart DH (1996) Urinary cytokines during intravesical bacillus Calmette-Guerin therapy for superficial bladder cancer: processing, stability and prognostic value. *J Urol* **155**: 477–482

Erton M, Ilker Y and Akdas (1996) Carcinoma in situ and treatment options. *Int Urol Nephrol* **28**: 33–42

Esrig D, Freeman J, Stein J and Skinner D (1997) Early cystectomy for clinical stage T1 transitional cell carcinoma of the bladder. *Semin Urol Oncol* **15**: 154–160

Hermann GG, Petersen KR, Steven K and Zeuthen J (1990) Reduced LAK cytotoxicity of peripheral blood mononuclear cells in patients with bladder cancer: decreased LAK cytotoxicity caused by a low incidence of CD56+ and CD57+ mononuclear blood cells. *J Clin Immunol* **10**: 311–320

Hurle R, Losa A, Ranieri A, Graziotti P and Lembo A (1996) Low dose Pasteur bacillus Calmette-Guerin regimen in stage T1, grade 3 bladder cancer therapy. *J Urol* **156**: 1602–1605

 © Cancer Research Campaign 1999

Jackson AM, Alexandroff AB, McIntyre M, Esuvaranathan K, James K and Chisholm GD (1994) Induction of ICAM 1 expression on bladder tumours by BCG immunotherapy. *J Clin Pathol* **47**: 309–312

Jackson AM, Alexandroff AB, Kelly RW, Skibinska A, Esuvaranathan K, Prescott S, Chisholm GD and James K (1995) Changes in urinary cytokines and soluble intercellular adhesion molecule-1 (ICAM-1) in bladder cancer patients after bacillus Calmette-Guerin (BCG) immunotherapy. *Clin Exp Immunol* **99**: 369–375

Kaempfer R, Gerez L, Farbstein H, Madar L, Hirschman O, Nussinovich R and Shapiro A (1996) Prediction of response to treatment in superficial bladder carcinoma through pattern of interleukin-2 gene expression. *J Clin Oncol* **14**: 1778–1786

Koss LM (1973) Tumours of the urinary bladder. In *Atlas of Tumour Pathology*, Armed Forces Institute of Pathology, (eds) pp ???

Kurth K (1997) Diagnosis and treatment of superficial transitional cell carcinoma of the bladder: facts and perspectives. *Eur Urol* **31**: 10–19.

Kurth K, Tunn U, Ay R, Schroder F, Pavone-Macaluso M, Debruyne F, ten Kate F, de Pauw M and Sylvester R (1997) Adjuvant chemotherapy for superficial transitional cell bladder carcinoma: long-term results of a European Organization for Research and Treatment of Cancer randomized trial comparing doxorubicin, ethoglucid and transurethral resection alone. *J Urol* **158**: 378–384

Lamm DL and Torti FM (1996) Bladder cancer, 1996. *CA Cancer J Clin* **46**: 93–112

Lapham R, Ro J, Staerkel G and Ayala A (1997) Pathology of transitional cell carcinoma of the bladder and its clinical implications. *Semin Surg Oncol* **13**: 307–318

Larson EL, Iscove NN and Coutinho A (1980) Two distinct factors are required for induction of T-cell growth. *Nature* **283**: 664–666

Mack D and Frick J (1995) Five-year results of a phase II study with low-dose bacille Calmette-Guerin therapy in high-risk superficial bladder cancer. *Urology* **45**: 958–961

Madsen M, Johnsen HE, Hansen PW and Christiansen SE (1980) Isolation of human T and B lymphocytes by E-rosette gradient centrifugation. Characterization of the isolated sub-populations. *J Immunol Methods* **33**: 323–336

Maes H, Taper H and Cocito C (1995) Alteration of the immune response during cancer development and prevention by administration of a mycobacterial antigen. *Scand J Immunol* **41**: 53–64

Molto L, Alvarez-Mon M, Carballido J, Olivier C, Gimeno F and Manzano L (1995) Use of intracavitary interferon alpha 2b in the prophylactic treatment of patients with superficial bladder cancer. *Cancer* **75**: 1720–2726

Molto L, Carballido J, Manzano L, Reyes E, Olivier C and Alvarez-Mon M (1997) Prophylactic intracavitary treatment with interferon alpha increases interferon gamma production by peripheral blood mononuclear cells in patients with superficial transitional cell carcinoma of the bladder. *Br J Cancer* **75**: 1849–1853

Nadler RB, Catalona WJ, Hudson MA and Ratliff TL (1994) Durability of the tumor-free response for intravesical bacillus Calmette-Guerin therapy. *J Urol* **152**: 367–373

Nicks M, Otto M, Busova B and Stefanovic J (1990) Quantification of proliferative and suppressive responses of human T lymphocytes following ConA stimulation. *J Immunol Methods* **126**: 263–271

O'Donnell MA and DeWolf WC (1995) Bacillus Calmette-Guerin immunotherapy for superficial bladder cancer. New prospects for an old warhorse. *Surg Oncol Clin N Am* **4**: 189–202

Pansadoro V, Emiliozzi P, Defidio L, Donadio D, Florio A, Maurelli S, Lauretti S and Sternberg CN (1995) Bacillus Calmette–Guerin in the treatment of stage T1 grade 3 transitional cell carcinoma of the bladder: long-term results. *J Urol* **154**: 2054–2058

Patard JJ, Chopin DK and Boccon Gibod L (1993) Mechanisms of action of bacillus Calmette-Guerin in the treatment of superficial bladder cancer. *World J Urol* **11**: 165–168

Picker LJ, Terstappen LWMM, Rott LS, Streeter PR, Stein H and Butcher EC (1990) Differential expression of homing-associated adhesion molecules by T cell subsets in man. *J Immunol* **145**: 3247–3255

Prescott S, James K, Hargreave TB, Chisholm GD and Smyth JF (1992) Intravesical Evans strain BCG therapy: quantitative immunohistochemical analysis of the immune response within the bladder wall. *J Urol* **147**: 1636–1642

Pryor K, Goddard J, Goldstein D, Stricker P, Russell P, Golovsky D and Penny R (1995) Bacillus Calmette-Guerin (BCG) enhances monocyte- and lymphocyte-mediated bladder tumour cell killing. *Br J Cancer* **71**: 801–807

Ratliff TL, Ritchey JK, Yuan JJ, Andriole GL and Catalona WJ (1993) T-cell subsets required for intravesical BCG immunotherapy for bladder cancer. *J Urol* **150**: 1018–1023

Rosenbaum RS, Park MC and Fleischmann J (1996) Intravesical bacille Calmette-Guerin therapy for muscle invasive bladder cancer. *Urology* **47**: 208–211

Satoh M, Seki S, Hashimoto W, Ogasawara K, Kobayashi T, Kumagai K, Matsuno S and Takeda K (1996) Citotoxic γδ or αβ T cells with a Natural Killer cell marker, CD56, induced from human peripheral blood lymphocytes by a combination of IL-12 and IL-2. *J Immunol* **157**: 3886–3892

Takashi M, Wakai K, Ohno Y, Murase T and Miyake K (1995) Evaluation of a low-dose intravesical bacillus Calmette-Guerin (Tokyo strain) therapy for superficial bladder cancer. *Int Urol Nephrol* **27**: 723–733

Thanhauser A, Bohle A, Schneider B, Reiling N, Mattern T, Ernst M, Flad HD and Ulmer AJ (1995) The induction of bacillus-Calmette-Guerin-activated killer cells requires the presence of monocytes and T-helper type-1 cells. *Cancer Immunol Immunother* **40**: 103–108

Turner J and Dockrell HM (1996) Stimulation of human peripheral blood mononuclear cells with live *Mycobacterium bovis* BCG activates cytolytic CD8+ T cells in vitro. *Immunology* **87**: 339–342

Verhagen A, Mackay IR, Rowley M and Tymms M (1990) Comparison of augmentation of human natural killer cell cytotoxicity by interferon-alpha subtypes. *Nat Immun Cell Growth Regul* **9**: 325–333

Walker PR, Ohteki T, Lopez JA, MacDonald HR and Maryanski JL (1995) Distinct phenotypes of antigen-selected CD8 T cells emerge at different stages of an in vivo immune response. *J Immunol* **155**: 3443–3452

Witjes J (1997) Current recommendations for the management of bladder cancer. Drug therapy. *Drugs* **53**: 404–414

Zlotta A, Drowart A, van Vooren JP, Simon J, Schulman CC and Huygen K (1994) Evolution of cellular and humoral response against tuberculin and antigen 85 complex during intravesical treatment with BCG of superficial bladder cancer. *Acta Urol Belg* **62**: 63–68

*© Cancer Research Campaign 1999*
